# A rare inflammatory myofibroblastic tumor appearing both inside and outside the heart

**DOI:** 10.1186/s40001-024-01710-0

**Published:** 2024-02-17

**Authors:** Jiarong Li, Jijia Liu, Xingwang Yao, Jinfu Yang

**Affiliations:** 1 Department of Cardiovascular Surgery, The Second Xiangya Hospital, Central South University, Middle Renmin Road 139, Changsha, 410011 China; 2 Clinical Nursing Teaching and Research Section, The Second Xiangya Hospital, Central South University, Changsha, 410011 China

**Keywords:** Cardiac tumor, Inflammatory myofibroblastic tumor, cIMT, Rare, Surgical management

## Abstract

**Background:**

Inflammatory myofibroblastic tumor (IMT) is an uncommon cardiac tumor that primarily affects infants, children, and young adults. While complete surgical resection generally leads to a favorable prognosis, accurate diagnostic tests remain limited.

**Case presentation:**

We describe the case of a 26-year-old female who had a dual tumor inside and outside the heart and was misdiagnosed by echocardiography and MRI. We also review 71 cases of cardiac IMTs from the literature regarding their epidemiology, clinical presentation, and outcome.

**Conclusion:**

Early detection of this rare disorder is essential for optimal surgical management.

## Introduction

Inflammatory myofibroblastic tumor (IMT) is an exceptionally rare benign tumor with an unknown etiology, consisting of plasma cells, lymphocytes, histiocytes, and vascular tissue. Since it was first discovered in 1939 by Brunn [1], very few cases are reported and especially the cardiac origin. Although rhabdomyomas, myxomas, and other mesenchymal tumors are more commonly encountered intracardiac tumors, IMTs represent a distinct subset within cardiac neoplasms. [2, 3] Consequently, determining the epidemiological trends associated with this specific type of IMT has posed a significant challenge. The first cIMT case was described in 1975 [4], describing a tumor located in the left atrium of a pediatric patient. Subsequent to this report, there have been sporadic case accounts and limited literature reviews concerning cardiac IMTs [5–18]. These tumors were previously believed to primarily affect children and young adults. In this report, we present an intriguing case study of a 26-year-old female who experienced chronic chest tightness and had a large IMT occupying a large portion of the atrial septum and invading the aortic sinus. Additionally, a similar tumor was observed within the wall of the ascending aorta.

## Case report

**Fig. 1:**
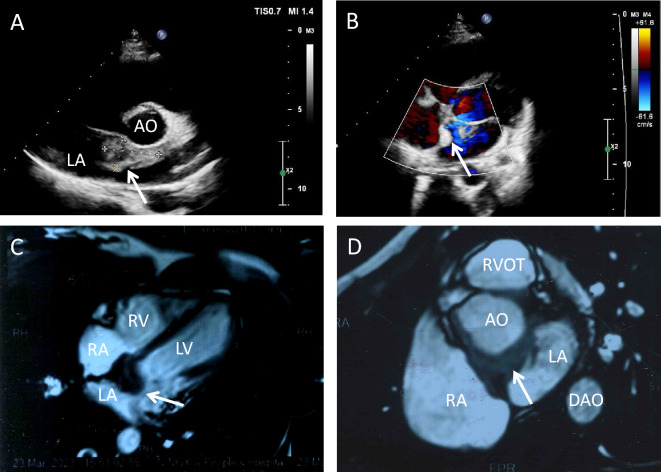
**A** Echocardiography and color Doppler blood flow image (**B**) from left atrium angle, white arrow indicates the tumor. **C** Nuclei magnetic resonance image from long axis, white arrow indicates the tumor. **D** Nuclei magnetic resonance image from aortic valve plane. White arrow indicates the tumor. *LA* left atrium, *AO* aortic, *RA* right atrium, *LV* left ventricle, *RV* right ventricle, *DAO* descending aorta, *LCS* left coronary sinus, *RCS* right coronary sinus, *NCS* left coronary sinus

A 26-year-old female was referred to our department with dyspnea and mild chest oppression on exertion for more than 9 months, worsened for 1 month. She had no family history of hypertension, no history of chest trauma or other cardiac health comorbidities. Upon physical examination, a soft blowing murmur was auscultated in the second costal margin of the left sternum. No other significant clinical findings were noted during the examination. The trans-thoracic echocardiogram (Fig. 1A and B) pointed out a marked intra-atrial septal mass with irregular margins. The electrocardiogram demonstrated first-degree atrioventricular block. The cardiac magnetic resonance (CMR) demonstrated invasion of the left atrium and aortic sinus by an abundant solid tissue in the atrial septal with a clear boundary (Fig. 1C and D).

**Fig. 2:**
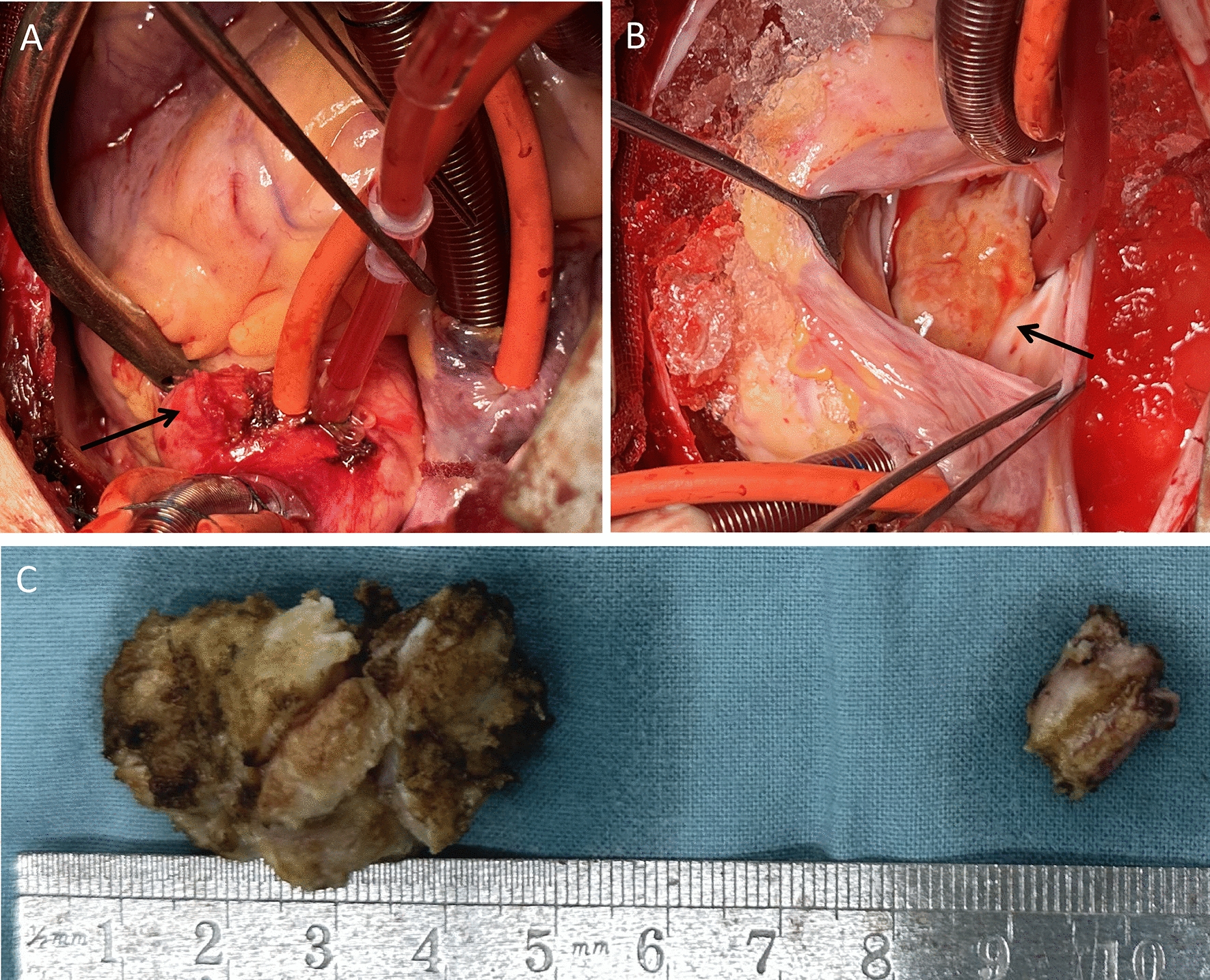
**A**, **B** Surgical field of the open-heart surgery, black arrowhead indicates the tumor inside of the heart, black arrow indicates the tumor outside of the heart. **C** Measuring the diameter of both the tumor

Upon admission, the patient underwent open-heart surgery. A regular median sternotomy incision was made, providing access to the surgical area. A 15 × 10 mm solid mass was found at the lateral to the ascending aorta near the root (Fig. 2A). With cannulation of the aorta superior to the mass, and separate cannulas in the superior vena cava and inferior vena cava, cardiopulmonary bypass was established as per standard protocol. Following the opening of the right atrium, the mass was found to locate inside the atrial septal, with body length 45 × 30 mm, exhibited a grayish-yellow color, and had a lobulated structure with a firm and smooth surface, upper limit reached the roof of left atrium and invasive to posterior aortic sinus and left outflow tract (Fig. 2B). Both the masses were completely removed (Fig. 2C) and a bovine pericardial patch was used to rebuild the atrial septum and the posterior aortic sinus, another patch was used to fix the ascending aorta.

**Fig. 3:**
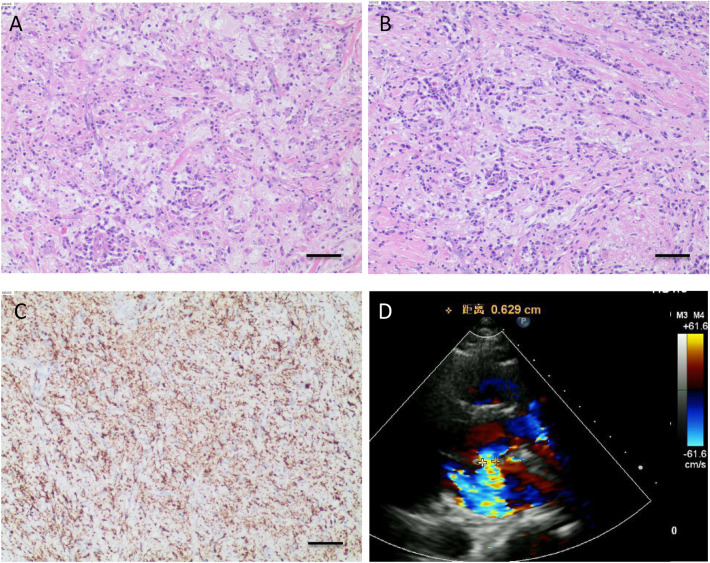
HE staining (**A** and **B**) and CD68 staining (**C**) of the tumorous tissue confirm the diagnosis of inflammatory myofibroblastic tumor, scale bar = 20 μm. The red-stained long strip-shaped indicated cardiomyocytes, blue-stained spindle-shaped indicated myofibroblasts, other blue-stained cells indicated plasma cells or macrophages. Echocardiography 8 months post-operatively (**D**) shows no hemodynamic abnormality

Cosmetically the two masses should be of the same origin, but it was not possible to determine the nature of the masses visually, pathologic examination and anaplastic lymphoma kinase (ALK) gene testing were subsequently performed. In both sections of the two tumors, a large amount of proliferative vitreous fibrous tissue infiltrated by lymphoplasmacytic cells and histiocytes and scattered adipocytes were seen under the microscope, plump spindle cells were arranged in a disorganized manner and were seen surrounding myocytes in a focal manner. Notably, a substantial influx of chronic inflammatory cells, including plasma cells (Fig. 3A and B), macrophages (CD68 + , Fig. 3C) are also present, suggesting fibrous histiocytoma-like hyperplasia. Immunohistochemistry (IHC) showed CK(-), CD20(+), CD38(+), SMA(+), DES(+), CD38(+), CD138(+), IgG4(+), CD34(-), S-100(+), P53(-). ALK staining through VENTANA(D5F3) method resulted positive in the positive controlling group but was negative in negative controlling group. To further confirm the absence of ALK-1 translocation, ALK break-apart fluorescence in situ hybridization (FISH) probe analysis was performed, resulting in a negative finding.

The postoperative outcomes were satisfactory. Three days following the surgery, echocardiography revealed complete removal of the mass, with normal functioning of the aortic valve. The patient did not experience any significant discomfort and was discharged from the hospital after 2 days. During the 1-month and 6-month follow-up period, no adverse events or unforeseen complications were reported. The echocardiography at 8-month post-operatively showed no hemodynamic abnormality (Fig. 3D), no metastasis and recurrence were found.

## Discussion

Cardiac tumors are a rare occurrence in children, with an incidence ranging from 0.01% to 0.32% [19]. Most of these tumors are hamartomas and benign neoplasms, including rhabdomyomas, myxomas, teratomas, and fibromas [3, 20]. However, IMT of the heart is extremely rare, since B. Xu’s review in 2014 [5], and currently, there are only 71 cases documented in the English literature (Table 1). The case we reported here is the first one which characterized as dual IMT appear both inside and outside the heart. It is noteworthy that both tumors were located in the interatrial septum and the vessel wall, a finding not described in any previous reports. This makes us realize that cardiac IMT does not only involve myocardium and valves, it further enhances the diversity of sites involved in IMT and the importance of early diagnosis of cardiac IMT.

**Table 1:** Cases reported in recent years

First author (year)	Patient age	Patient sex	Symptom	Previous medical history	Tumor site	Other findings	Tumor maximal dimension	Treatment	Known follow-up
Bin Xu [5]	9 y	F	Lost conscious	None	AV	None	1.5 cm	Autopsy	Sudden death
Min Bao [6]	2 m	F	Fever, cough	None	MV, LA	None	2.0 cm	Resection	Not mentioned
Wenzong Luo [7]	55 y	F	Chest pain	None	LVOT	None	3.0 cm	Resection and AV reconstruction	2 y, NED
Aleksejus Zorinas [8]	43 y	F	Heart failure	Multi neoplasms	LVOT	None	3.0 cm	Resection	9 m, Dead
Ming-dan Deng [9]	45 y	F	None (found by body examination)	None	IVS	None	4.0 cm	Resection	12 m, NED
Aitizaz U Syed [10]	4 m	F	PE	None	RA	None	3.0 cm	Resection	6 m, NED
Tommaso D'Angelo [11]	9 y	M	Microhematuria, fever, dyspnea, lethargy	None	RV, PV	None	3.8 cm	Resection	2 m, NED
Diego Monzón Díaz [12]	66 y	M	Headache	HBP, AF	RV	None	3.0 cm	Resection	Not mentioned
Yasemin Nuran Donmez [13]	3 m	M	Poor feeding, shortness of breath	None	Intrapericardial	PT	6.0 cm	Resection	2 y, NED
Shoken Suzuki [14]	25 y	M	None	None	LVOT	None	6.0 cm	Autopsy	Sudden death
Niranjan Vijayakumar [15]	8 y	F	Right thigh and leg pain, fever	None	MV, LV, AV	Systemic embolism	N.A	Resection	3 m, NED
Weiwei Zhu [16]	7 y	M	Syncope	None	MV	Acute cerebral infarction	2.3 cm	Resection, mitral valvuloplasty in following 8th month	6 y, NED
Lauren M. McKinney [17]	2.5 m	F	None (Detected by prenatal ultrasound)	None	RA	TOF with absent PV	N.A	Resection	13 m, NED
B. M. Soares [18]	4 m	M	Facial, cervical, and upper thoracic edema	None	RA	SVCS, PE	4.0 cm	resection	36 m, NED

*AV* aortic valve, *F* female, *IVS* intraventricular septum, *LA* left atrium, *LVOT* left ventricle outflow tract, *m* month, *M* male, *MV* mitral valve, *N/A* not available, *NED* no evidence of disease, *PE* pericardial effusion, *PT* pericardial tamponade, *PV* pulmonary valve, *RA* right atrium, *RV* right ventricle, *SVCS* superior vena cava syndrome, *TOF* tetralogy of Fallot, *y* year

Cardiac IMT usually be benign, most of the cases did not show any syndromes, and the presence always to be hidden. Most of the patients did not seek help until the tumor grew and blocked the valve structure, outflow tract, or coronary orifice [21–25].

Histopathology plays a critical role in diagnosing IMT, with a distinctive histological pattern characterized by spindle-shaped myofibroblastic proliferation and a chronic inflammatory infiltrate. The histological section through the tumor shows the spindle-shaped myofibroblasts and the scattering of small lymphocytes in the stroma that are characteristic of this tumor. Although there are some similarities with myosarcomas [26], cIMTs exhibit less pleomorphism, atypia, and fewer mitotic figures.

The predominant differential diagnoses for the observed histologic appearance include cardiac IMT and IgG4-related disease. However, in comparison to IgG4-related disease, which can erode muscle, IMT has not been widely reported to involve cardiac muscle. In the IHC result of this case, we found a sporadic IgG4 positive, but according to the reported IgG4 cardiac tumor cases [27] and review [28], IgG4-related cardiac disease is extremely rare to see, its histological appearance commonly includes features such as storiform fibrosis, obliterative phlebitis, an increased presence of IgG4-positive plasma cells crowded with significant numbers of lymphocyte. Although the reported tumor site already expanded from cardiac muscle to vessel structure [29], the above characteristics are not supportive for matching our case here.

Cardiac IMTs occur predominantly in children and young adults, according to B. Xu’s review and our summary, the mean patient age of presentation is about 16.9 years. Among the documented cases, 26 out of 71 patients (36.6%) were 1 year of age or younger, while 24 patients (33.8%) fell within 1 to 20-year age. The majority of these cases were characterized by polypoid lesions originating from the endocardium, protruding into the cardiac cavity. The cases invasive in the right heart system (RA, RV, PA, PV) versus left heart system (LA, LV, MV, AV) is ratio 37:27, only two cases just seen in IVS, and only one just found in pericardium, no case ever reported tumor inside and outside heart at the same time like our case. 28 of 71 cases found valve affected, implies that tumor sources include myogenic and fibrous origin. In general, cardiac IMTs are considered benign, with a low rate of metastasis. However, their dangerous nature requires special attention. Of the 56 cases with follow-up data available, 12 resulted in death, for a mortality rate of 21.4%. Death was attributed to sudden death due to the tumor or perioperative complications.

In the majority of cases, the tumor is removed without recurrence or other cardiac complications. Pharmacological intervention is not commonly used, and health care professionals should attempt to remove the tumor as completely as possible. Surgical resection is the most common treatment approach for cIMT. Comparing to a recurrence rate 5% of total reported case by year 2012 [30], However, among the 36 patients who underwent complete resection and had subsequent clinical follow-ups, 29 remained asymptomatic with no evidence of disease/recurrence, resulting in a recurrence rate of 19.4%. On the other hand, in the four patients who only received partial resection, a recurrence or progression of the tumor was documented in all cases, leading to a 100% recurrence rate. Additionally, two out the four patients who underwent partial resection eventually died of the disease. It is crucial to highlight the importance of complete surgical resection in achieving more favorable outcomes and reducing the risk of recurrence or disease progression.

Currently, there are no biochemical markers indicative of the prognosis of cardiac IMTs. Immunohistochemistry analysis often shows positive staining for vimentin, smooth muscle actin (SMA), and muscle-specific actin (MSA) in the majority of cases, while ALK staining varies in positivity [5, 31]. Historically, the association between ALK gene and the tumor has been considered. ALK reactivity has been reported to be linked to the local recurrence, but not the distant metastasis [32]. But compared to 40–60% of positive rate in the extra cIMTs, only 10% of cIMTs showed ALK-1 overexpression within IHC [5, 31]. More cases are still needed to judge the diagnostic value of ALK-1 in cIMT, with this reason, in contrast to B. Xu’s review, we did not list the histological and immunohistochemistry features in Table 1. An alternative approach to studying cIMTs involves genetic testing to identify specific genetic abnormalities. This includes examining the 2p23 locus for clonal ALK gene rearrangements and detecting ALK gene fusion with proto-oncogenes, as well as ALK overexpression and other potential genetic alterations like EML4-ALK inversion [33]. This genetic testing can provide valuable information about the molecular characteristics of cardiac IMTs and aid in their diagnosis and management [34].

## Data Availability

Data are available from the corresponding author upon reasonable request.
